# A novel processing approach for free-standing porous non-oxide ceramic supports from polycarbosilane and polysilazane precursors

**DOI:** 10.1016/j.jeurceramsoc.2015.03.009

**Published:** 2015-09

**Authors:** Thomas Konegger, Rajesh Patidar, Rajendra K. Bordia

**Affiliations:** a Department of Materials Science and Engineering, Clemson University, 161 Sirrine Hall, Clemson, SC 29634, USA; b Institute of Chemical Technologies and Analytics, Vienna University of Technology, Getreidemarkt 9/164-CT, 1060 Vienna, Austria; c Department of Mechanical Engineering, Indian Institute of Technology Gandhinagar, India

**Keywords:** Polymer-derived ceramics, Polycarbosilane, Polysilazane, Support, Porosity

## Abstract

In this contribution, a low-pressure/low-temperature casting technique for the preparation of novel free-standing macrocellular polymer-derived ceramic support structures is presented. Preceramic polymers (polycarbosilane and poly(vinyl)silazane) are combined with sacrificial porogens (ultra-high molecular weight polyethylene microbeads) to yield porous ceramic materials in the Si—C or Si—C—N systems, exhibiting well-defined pore structures after thermal conversion.

The planar-disc-type specimens were found to exhibit biaxial flexural strengths of up to 60 MPa. In combination with their observed permeability characteristics, the prepared structures were found to be suitable for potential applications in filtration, catalysis, or membrane science.

## 1 Introduction

Porous non-oxide ceramics such as SiC and Si_3_N_4_ are interesting materials due to their ability to bear high mechanical stresses, high temperatures, as well as corrosive environments, suggesting their suitability as support structures for catalysis [1] or membrane applications [2–4]. However, the generation of these macroporous structures typically requires partial sintering of particulates at high temperatures.

An alternative to conventional sintering-based consolidation approaches is the use of the polymer pyrolysis technique, in which non-oxide ceramics are produced through pyrolytic conversion of Si-based precursors such as polysilazanes or polycarbosilanes. This method allows for a high flexibility during processing, high purity of products, and decreased temperature requirements during consolidation [5].

Porous polymer-derived ceramics can be obtained through a combination of the polymer pyrolysis route with a variety of pore forming techniques, including replica forming, direct foaming, or by using sacrificial fillers [6,7]. The use of sacrificial fillers such as polymethyl methacrylate (PMMA) [8], polystyrene (PS) [9], or polyethylene (PE) [10], which are removed by thermal decomposition during the thermal conversion of the preceramic polymers, allows for a high tailorability and reproducibility of the pore structure, which is highly desired for potential applications with reproducible strength and permeability properties [11]. In the majority of reports, compaction processes (cold- or warm-pressing) were used for the consolidation of the precursor/porogen mixtures. While the use of liquid precursors in combination with sacrificial fillers has been described in the literature, the applied techniques (e.g., micromolding [9]) were strongly limited in terms of sample size and shape.

In this paper, we present a new, versatile technique to obtain porous, non-oxide, polymer-derived ceramics by following a sacrificial filler approach, employing a pressureless casting/cross-linking process with subsequent conversion of the precursors at low temperatures, thus facilitating the preparation of free-standing, macroscopic specimens with relative ease. This process is also expected to be easily scalable and capable of producing complex shapes. The objective of this work is the demonstration of the feasibility of this technique for different preceramic polymers, yielding porous non-oxide ceramics with homogeneous pore distributions, as well as strength and permeability characteristics facilitating potential applications as catalyst or membrane supports.

## 2 Experimental procedure

In this study, two commercially available liquid preceramic polymers were used. Allyl hydrido polycarbosilane (*PCS*; SMP-10, Starfire, USA) was used as a precursor for Si-C-based ceramics, and poly(vinyl)silazane (*PSZ*; HTT1800, AZ Electronic Materials, USA) was used as a precursor for Si-C-N-based ceramics. In both cases, 1 wt% of a radical initiator (Dicumyl peroxide, 99%, Acros Organics, USA) was added to promote cross-linking. An ultra-high molecular weight polyethylene powder (*UHMW-PE*, Mipelon PM-200, Mitsui Chemicals America, USA; molecular weight of 1.8 × 10^6^ g mol^−1^) with a mean particle size of 10 μm was used as sacrificial filler at a volume fraction of 30%. Before use, the polyethylene powder was dried in vacuum at 50 °C for 12 h.

Due to the reactivity of the polymer precursors with air and water, handling of the materials was conducted in inert atmosphere, using a glove-box. After fully dissolving the radical initiator in the polymers by magnetic stirring, and subsequent vacuum degassing, corresponding amounts of polymer and filler were mixed by magnetic stirring in vacuum for a minimum of 30 min. The obtained viscous mixture was cast in polydimethylsiloxane moulds (Mold Max XLS II, Smooth-On Inc., USA) with cylindrical cavities (diameter of 18 mm). Subsequently, the mixture was cross-linked at 105 °C for 16 h in flowing N_2_. After demolding, a thermal post-curing step using the same conditions was carried out. The top and bottom surfaces of the cross-linked discs were ground to a 2000 grit finish. The specimens were fully crosslinked and pyrolyzed in high-purity flowing Ar (PCS-derived samples) or N_2_ (PSZ-derived samples) atmosphere. The thermal treatment included an initial heating rate of 1 K min^−1^ (Lindberg Blue M HTF5534C, Thermo Scientific, USA) to the cross-linking temperature (PCS: 2 h hold at 130 °C and 1 h hold at 250 °C; PSZ: 2 h hold at 130 °C). Following this step, a heating rate of 0.5 K min^−1^ was used to heat the samples to the pyrolysis temperature (4 h hold at 800 °C).

The pore structure of the pyrolyzed ceramic discs with final diameters of around 14 mm and heights of around 2 mm was investigated by scanning electron microscopy (*SEM*; S4800, Hitachi, Japan). The bulk density was calculated from the sample weight and geometric dimensions. The total porosity and the pore opening size distribution were measured by mercury intrusion porosimetry (PoreMaster 33, Quantachrome, USA). The specific surface area was determined by N_2_ adsorption employing the Brunauer–Emmett–Teller (BET) method (ASAP 2010, Micromeritics, USA). Before conducting the adsorption runs, the samples were degassed in vacuum at 200 °C for 24 h. To evaluate the mechanical strength of the samples, a biaxial flexural test employing a ball-on-three-balls (B3B) setup [12] was used with stainless steel balls of 9.525 mm diameter. A Poisson's ratio of 0.2 was assumed for both sets of samples. In order to estimate strength variability, 15 samples were tested for each composition.

Permeability of the pyrolyzed supports (8 samples per composition) was determined by a capillary flow porometer (CFP-1100-AEXS, Porous Materials, Inc., USA) at a pressure difference Δ*p* up to 1 bar using Darcy's law, employing air as the fluid.

## 3 Results and discussion

The development and successful implementation of a low-pressure casting technique allowed for the reproducible production of large numbers of polymer-derived ceramic samples without the need to use pre-crosslinked precursor materials or time-consuming and shape limited consolidation techniques such as warm-pressing.

After cross-linking, bubble-free specimens were obtained, which were readily formable by cutting and grinding, thus yielding planar disc-shaped specimens. In addition to low heating rates during cross-linking, thorough degassing during mixing was found to play a major role for the successful generation of dense, cross-linked samples. While a vacuum level of around 10 mbar was found to be sufficient for PSZ-containing samples, bubble-free PCS-containing samples could only be obtained after degassing at vacuum levels below 1 mbar, due to the higher viscosity of this precursor liquid.

**Fig. 1:**
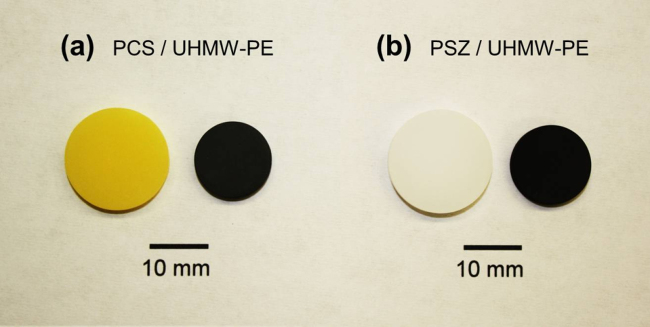
Appearance of PCS (a) or PSZ (b) specimens containing UHMW-PE before (left) and after pyrolytic conversion (right).

Pyrolytic conversion in inert atmosphere resulted in the generation of porous ceramic samples without macroscopic cracks or significant shape distortion (e.g., warping) (Fig. 1).

**Fig. 2:**
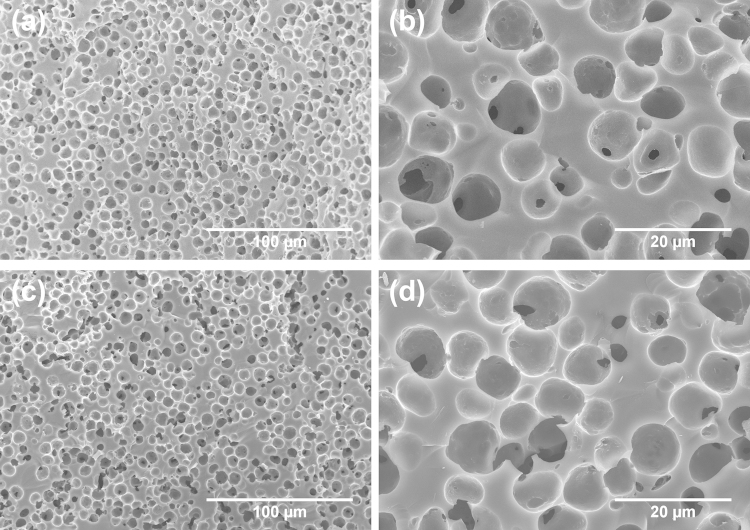
SEM micrographs of the fracture surface of porous ceramic supports obtained from UHMW-PE-containing PCS (a, b) or PSZ (c, d).

After pyrolytic conversion, porous amorphous ceramics in the Si—C system (PCS) or the Si—C—N system (PSZ) were obtained. No residue from the sacrificial filler materials was observed in the pore voids. The samples contain an open pore network consisting of circular pores which are interconnected by smaller pore windows (Fig. 2). Owing to the close proximity of densities of the preceramic polymers and the sacrificial fillers (PCS: 0.97 g cm^−3^, PSZ: 1.02 g cm^−3^, UHMW-PE: 0.94 g cm^−3^), no segregation of the pore former was observed during casting or cross-linking, thus allowing for a homogeneous, albeit random distribution of pores in the final material.

In both materials systems, the average major pore diameters are 7–8 μm, corresponding to the initial porogen size of 10 μm. This is an expected result taking into account typical linear shrinkages of 24–25% during pyrolytic conversion [13].

**Fig. 3:**
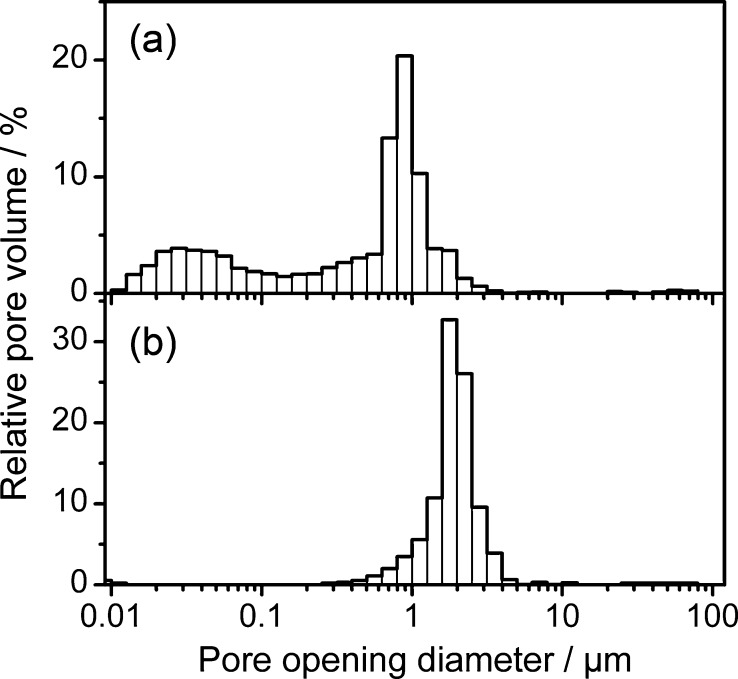
Pore opening size distribution of porous ceramic supports derived from UHMW-PE-containing PCS (a) or PSZ (b), obtained by mercury intrusion porosimetry.

An investigation of the pore structure by mercury intrusion porosimetry showed a strong dependence of the choice of preceramic polymer on the resulting pore opening diameters (Fig. 3).

While PSZ-derived samples exhibited a narrow, monomodal pore opening size distribution with a median diameter of 1.9 μm, PCS-derived samples showed a shift towards smaller pore openings as well as a tailing of pore openings into the mesopore range (<50 nm), resulting in a median pore opening size of 0.7 μm. The smaller pore window size can potentially be explained by differences in the wetting characteristics of the porogen particles by the polymers (i.e., a better wetting resulting in a shift towards smaller pore opening sizes). While the high degree of ink-bottle type porosity (large pores connected by small pore openings) in the PCS samples can be assumed to the main factor for the apparent shift towards lower pore diameters, the generation of mesopores in the PCS-derived ceramic backbone structure also potentially contributes. It is known that mesoporosity generated during pyrolysis, in inert atmosphere, generally disappears in PSZ-derived materials at temperatures exceeding 700 °C [14], while micro- and mesopores have been reported to be retained in cross-linked PCS-derived materials up to 800 °C [15]. Differences in the specific surface areas between the different materials give further evidence of the presence of larger amounts of sub-μm porosity in the PCS-derived material, resulting in higher specific surface areas of the PCS-derived material in comparison to the PSZ-derived sample (Table 1).

While having the potential drawback of being limited to lower maximum porogen loads and thus final porosities in comparison to compaction-based consolidation techniques due to the required castability of the precursor/porogen mixtures, strength and permeability properties of the generated materials are of major interest for potential applications as support structures in membrane or catalysis applications. Depending on the preceramic polymer used, an initial porogen content of 30Vol.% resulted in final porosities in the range of 40–50% (Table 1), which is in accordance with general recommendations for membrane support materials [16]. The discrepancy between the initial porogen content and the resulting final porosities can potentially be explained by two factors: (a) the presence of gas bubbles present on the surface of porogen particles, either as a result of incomplete wetting during mixing or formed during thermal treatment; and (b) additional porosity generated in the matrix during pyrolysis. In the case of PCS-derived samples, the reduction in pore opening size results in a significant decrease in gas permeability, around 75% lower than the permeability of analogously prepared PSZ-derived samples. For supports with a typical thickness *x* of 2.5 mm as used in this work, gas permeances *P* of 10^−6^ to 10^−5^ mol m^−2^ Pa^−1^ s^−1^ can be derived from the obtained permeability values *k* with *P* = *k* μ^−1^ *x*^−1^, *μ* being the gas viscosity at 25 °C (*μ*_*air*_ = 18.5 × 10^−6^ Pas). These permeances are in the same order of magnitude as permeances of conventionally produced SiC membrane supports reported in the literature [4,17].

Due to the absence of extensive crack formation during the pyrolytic conversion, the samples demonstrated biaxial flexural strength values of up to 60 MPa, which can be considered adequate for many potential applications in membrane science (e.g., gas separation membranes) or catalysis. An increased cell window size, as found in samples derived from PSZ, did not result in decreased mechanical properties. A potential explanation of the lower mean strength of PCS-derived samples is the higher total porosity, albeit the domains of uncertainty of the present results do not permit a definite correlation between the two factors.

## 4 Conclusions

By implementing a straightforward mixing/casting/cross-linking procedure followed by low-temperature pyrolysis, porous non-oxide ceramics were successfully prepared from preceramic polymers with sacrificial fillers. Independent of pressure-assisted compaction techniques, the presented methodology allows for the preparation of bubble-free, dense cross-linked specimens and, subsequently, ceramic materials with a high flexibility in terms of materials composition, shape, and pore structure. This work demonstrates the feasibility of the proposed fabrication method to generate novel non-oxide, porous ceramic supports for catalysts or membranes. The processing approach is both scalable (in terms of size and volume) and capable of producing a variety of shapes. A variation of the content and the size of sacrificial filler particles can be expected to significantly affect the physical properties, thus allowing for a tailorability of the pore structure and, ultimately, a control of the permeation and strength characteristics.

## Figures and Tables

**Table 1:** Physical properties of planar disc-shaped polymer-derived ceramic supports. Both average properties and the range are reported, as well as the number of samples tested for each individual property.

	PCS/30 Vol% PE	PSZ/30 Vol% PE	No. of samples
Density (g cm^−3^)	1.14 ± 0.03	1.16 ± 0.02	15
Total porosity^a^ (%)	50.6 ± 1.3	42.0 ± 0.7	1
Specific surface area (m^2^ g^−1^)	2.50 ± 0.11	0.50 ± 0.04	1
Flexural strength, B3B (MPa)	47 ± 13	60 ± 16	15
Permeability (air) (10^−15^ m^2^)	2.5 ± 0.5	9.5 ± 1.5	8

a By mercury intrusion porosimetry; range is estimated based on density data.
